# The feasibility of an m-health educational programme (m2Hear) to improve outcomes in first-time hearing aid users

**DOI:** 10.1080/14992027.2020.1825839

**Published:** 2020-11-01

**Authors:** Melanie A. Ferguson, David W. Maidment, Rachel Gomez, Neil Coulson, Heather Wharrad

**Affiliations:** a National Institute for Health Research (NIHR) Nottingham Biomedical Research Centre, Nottingham, UK; b Hearing Sciences Section, Division of Clinical Neuroscience, School of Medicine, University of Nottingham, Nottingham, UK; c Queens Medical Centre, Nottingham University Hospitals NHS Trust, Nottingham, UK; d National Acoustic Laboratories, Australian Hearing Hub, Macquarie University, Sydney, Australia; e School of Sport, Exercise and Health Sciences, Loughborough University, Loughborough, UK; f Division of Rehabilitation, Ageing and Wellbeing, School of Medicine, University of Nottingham, Nottingham, UK; g School of Health Sciences, University of Nottingham, Nottingham, UK

**Keywords:** Multimedia, education, personalised, interactive, hearing aids, self-management, behaviour change, COM-B

## Abstract

**Objective:**

To (i) assess the delivery, accessibility, usability, acceptability, and adherence, and (ii) identify suitable outcome measures, for a mobile-enhanced multimedia educational programme (m2Hear) in first-time hearing aid users.

**Design:**

A prospective, single-centre feasibility study.

**Study sample:**

First-time hearing aid users (n = 59), recruited at their initial hearing assessment. Evaluations were made at 1-week and at 10–12 weeks post-hearing aid fitting.

**Results:**

m2Hear was most commonly accessed via tablets (42.3%). Usability was high for the System Usability Scale (88.5%), and the uMARS, particularly for the Information (M = 4.7), Functionality (M = 4.5) and Aesthetics (M = 4.2) subscales (maximum score = 5). Participant feedback was positive, with a high percent agreeing that m2Hear aided understanding of hearing aids (98%), held their interest (86%), improved confidence to use hearing aids and communicate (84%), and provided additional information to audiologist’s advice (82%). Learnings about practical hearing aid handling/maintenance skills and how to communicate with others were reportedly used equally in participant’s everyday lives. m2Hear was convenient to use, clear, concise and comprehensive. Outcome measures of social participation resulted in large effect sizes (Cohen’s *d* > 1.6).

**Conclusions:**

A theoretically-driven, personalised and co-designed educational m-health intervention is feasible and beneficial for use in the self-management of hearing loss and hearing aids.

## Introduction

Hearing aids are the main clinical intervention for hearing loss, however take-up and adherence of hearing aids are often poor; 1 in 3 people who would benefit from hearing aids fail to access them, and non-use can be high, up to 24% (Ferguson et al. 2017). The reasons for low take-up and adherence were highlighted in a James Lind Alliance Priority Setting Partnership for mild-moderate hearing loss as the fourth most important research priority (Henshaw et al. 2015). Reasons for non-use include difficulties inserting hearing aids, hearing aid maintenance and operation, and psychosocial factors, such as overly high expectations (Mccormack and Fortnum 2013; Bennett et al. 2018a), which all contribute to half (51%) of first-time hearing aid users having difficulties using their hearing aids (AOHL 2011).

High-quality information and readiness to accept hearing aids are key factors in successful individualised healthcare, and are identified in key UK national guidance documents (British Society of Audiology, 2016; NICE 2018). However, for hearing aid users, knowledge about hearing loss and hearing aids is often poor (Desjardins and Doherty 2009; Bennett et al. 2018b). They report receiving insufficient information (Kelly et al. 2013) and a need for more information (Laplante-Lévesque et al. 2013). Provision of information by audiologists to hearing aid users is often delivered verbally, but ∼50% of delivered information is forgotten within 6-weeks (Ferguson et al. 2015). A typical comment from a first-time hearing aid user is, “you get a lot of information… by the time you get home you’ve forgotten most of it”. This can be exacerbated by limited follow-up of patients, therefore in many cases all the important information that needs to be delivered for successful hearing aid use must be provided at the fitting appointment.

Patient-related knowledge is an essential part of health literacy, and is a core component of self-management and empowerment, particularly in people with long-term conditions (Bravo et al. 2015). Within audiology, a study to determine the structure of a self-management assessment tool conducted a factor analysis that indicated knowledge is one of the three cornerstones of successful self-management (Convery et al. 2019). This sits alongside actions (adoption and sustainability of behaviours to enhance adherence to a patient management intervention) and psychosocial behaviours (adoption and sustainability of behaviours leading to well-being and positive coping). The factor analysis identified two predictors of successful self-management that are modifiable (knowledge and self-efficacy), thus modifying and improving these factors can lead to greater action to self-manage hearing loss and hearing aids. In terms of empowerment, knowledge is one of five components included in Zimmerman’s theory of empowerment (knowledge, skills, self-efficacy, control, participation) (Zimmerman 1995). Although empowerment has not been systematically investigated within audiology, empowerment emerged as subtheme in a recent study of smartphone-controlled hearing aids, suggesting that giving the user the capacity to control and adjust their hearing aids can empower hearing aid users to successfully self-manage their hearing loss (Maidment, Ali, and Ferguson 2019).

To address the paucity of high-quality hearing-related knowledge available for hearing aid users, we developed and evaluated a home-delivered interactive multimedia educational programme (C2Hear; https://www.c2hearonline.com/). This was based on the concept of reusable learning objects (RLOs) that aimed to enhance patient benefit. RLOs include: (i) visual illustration of concepts, (ii) activity and engagement with content, and (iii) self-assessment. We have developed RLOs for first-time hearing aid users to address a range of practical (e.g. *how to insert hearing aids*) and psychosocial issues (e.g. *communication tactics*) relating to hearing aids and communication. Importantly, a participatory design was used that involved 32 hearing aid users and 44 hearing healthcare professionals to ensure the end-users’ “voice” was embedded in the development (Ferguson et al. 2018). The philosophy of the participatory design is to ensure that functionality and usability relating to the end users’ needs are aligned to the end-product (Jagosh et al. 2012; Convery et al. 2020).

The RLOs were evaluated in a clinically registered randomised controlled trial (RCT; ISRCTN11486888) of 203 first-time hearing aid users who received the RLOs at the hearing aid fitting appointment. The results were positive. There was significantly better knowledge of practical and psychosocial issues relating to hearing aids and communication, and significantly better practical hearing aid skills in the group receiving the RLOs (RLO+), with large clinical effect sizes (ES = 0.82–0.94). There was also significantly increased hearing aid use (15%) in the RLO + group compared to controls for patients who wore their hearing aids sub-optimally (ES = 0.83). In addition, the RLOs were rated as highly useful (9/10), and the majority (>80%) of participants agreed the RLOs were enjoyable, improved confidence and were preferable to written information. Take-up and use of the RLOs was also very high (78% and 94% respectively). In a second clinically registered RCT (NCT03912779), whereby the RLOs were offered at the hearing assessment appointment, self-efficacy for hearing aids improved significantly compared to a written booklet that covered similar information to the C2Hear RLOs, with a large clinical effect size (ES = 1.1) (Gomez and Ferguson 2020). Consistent with the earlier RCT, knowledge also improved significantly as a result of the RLOs, again with a large effect size (ES = 0.97). This study also indicated that the RLOs increased readiness for hearing rehabilitation, with a moderate effect size (ES = 0.5). Taken together, these studies demonstrate that the C2Hear RLOs can modify both knowledge and self-efficacy, which are components of health literacy, self-management and empowerment.

Despite the success of the RLOs, limitations included restricted interactivity and a “one size fits all” (i.e. non-personalised) approach because of the DVD platform that was used in the original development in 2012. This was because at that time, evidence from 1,235 people in Nottingham, UK showed that internet use was only 17% in the first-time hearing aid age group (70–74 years) and we wanted to maximise accessibility. The RCT showed that 32.9% accessed the RLOs through the internet (Ferguson et al. 2016). Since then, the use of the internet through mobile technologies (e.g. smartphone, tablets) in those over the age of 55 years has increased exponentially, from 40% in 2013 to 80% in 2019 (Deloitte 2019). The functionality of mobile technologies provides a platform that has been shown in other healthcare domains to increase user interactivity and engagement, as well as accessibility (Wang et al. 2014; Kim and Lee 2017). This ultimately enhances learning potential (Zhang et al. 2006; Bennett and Glasgow 2009), and enables tailoring of interventions to meet individual needs so that people can better manage their condition (Murray et al. 2016). Although some people voice concerns about the “digital divide” between younger and older people, the increasing digital literacy in the older population indicates that the delivery of electronic (e)- and mobile (m)-health technologies are appropriate for many of the typical first-time hearing aid user population. This has been shown in other hearing-related studies in this age group (Ferguson 2019; Maidment, Ali, and Ferguson 2019; Ng et al. 2017). Therefore, we have developed a theoretically driven, user-centred personalised intervention using mobile-enhanced RLOs (mRLOs) that goes beyond the “one size fits all” approach of C2Hear to enable greater personalised use and interactivity for hearing aid users to further improve patient outcomes.

The development and evaluation of our new m-health intervention was based on the COM-B model (Michie, van Stralen, and West 2011), a contemporary theoretical model that has informed the development and evaluation of complex behaviour change interventions in other health domains. The COM-B model addresses many issues of previous health behaviour models (Coulson et al. 2016) by proposing that for individuals to engage in a particular behaviour (B), such as hearing aid use, they must have physical and psychological capability (C), social and physical opportunity (O), and automatic and reflective motivation (M). These determinants of behaviour help to define what needs to change for a desired behaviour to occur (e.g. hearing aid use) or for an unwanted behaviour to cease (e.g. hearing aid non-use) (Edwards and Ferguson 2020). The COM-B model also provides a validated, integrative theoretical domains framework (TDF) that moves beyond the analysis of behaviour (Cane, O’Connor, and Michie 2012). This allows developers of interventions to identify which constructs (e.g. knowledge, skills, beliefs, emotions, intentions, reinforcement) are necessary to bring about change to inform the design and implementation of an intervention.

Our new m-health educational intervention, called m2Hear, was developed for use with mobile technologies, laptops and PCs via the internet in order to increase accessibility and interactivity. The mobile-enhanced intervention aimed to optimise hearing aid use (primary target behaviour), improve awareness of the consequences of hearing loss, and promote appropriate patient-directed self-management strategies to reduce participation restrictions associated with hearing loss. The development of m2Hear has been described elsewhere (Ferguson et al. 2019a, Maidment et al. 2020b) so only a brief description is given here.

The existing C2Hear RLOs were repurposed by dividing them into short learning segments that were on average 60 seconds in duration. This process was theoretically grounded, whereby each segment was classified according to the TDF, which links to a specific determinant of behaviour of the COM-B model. As such, we were able to identify the “active ingredients” of C2Hear that facilitate the target behaviour (hearing aid use) (Maidment et al. 2020b). The C2Hear RLOs covered most (12/14) of the TDF domains. Although domains associated with Capability (e.g. knowledge, physical skill, memory) were strongly represented in all RLOs, as would be expected for an educational intervention, there was also representation of domains relating to Opportunity and Motivation. For example, for Opportunity “Using the phone and other devices” had 82% in Environmental Context; for Motivation, “Getting to know your Hearing Aids” had 51% representation for Intentions. To complement the theoretical approach, an ecological method was adopted to provide labels for each mRLO to ensure the end-user perspective was included using a Think Aloud analysis. This is an established observational method to assess usability in product design and development (Fonteyn, Kuipers, and Grobe 1993). Sixteen hearing aid users viewed and simultaneously talked about the mRLOs in terms of what was important, relevant and valuable to them in relation to their hearing-related communication needs and experiences. This provided data that we used to label each mRLO with a specific question to enhance individualisation. For example, “What can I expect when wearing hearing aids for the first time?” and “What can I change to help me improve conversations?”, to enable hearing aid users to access specific content more readily.

The subsequent development of the m-health platform was iterative (*n* = 4 iterations), informing any content or usability modifications using a user-centered design with a panel of hearing aid users from the Think Aloud evaluation (*n* = 5). In addition, members of the project-specific patient and public involvement (PPI) panel formatively reviewed the intervention both in the lab and independently from home. These sessions were designed to assess user perceptions and interactions with the platform, as well as identify any potential problems.

On completion of the development process described, m2Hear was evaluated in a mixed-methods study. The main research question was: is it feasible for first-time hearing aid users to use a personalised educational intervention delivered through mobile technologies in their everyday life? The specific objectives of the research reported here were to:

1. Establish the feasibility of the m-Health intervention by evaluating delivery, accessibility, usability, adherence, and acceptability in first-time hearing aid users.
2. Identify suitable outcome measures to evaluate the effectiveness of the intervention in a future study (e.g. a randomised controlled trial).

## Methods

### Participants

Adult first-time hearing aid users were recruited following their hearing assessment appointment at the Nottingham Adult Audiology Service, Nottingham University Hospitals National Health Service (NHS) Trust. The inclusion criteria were (i) adults aged ≥18 years; (ii) never worn hearing aids; (iii) were familiar with smartphone technologies (e.g. owns a smartphone or tablet device, or uses one regularly), and (iv) had a good understanding of the English language in order to understand the mRLO content. The exclusion criterion was those who were unable to complete questionnaires without assistance due to age-related problems (e.g. cognitive decline or dementia).

## Study design and procedure

The design was a single centre, prospective, registered feasibility study (NCT03136718). Interested patients who met the eligibility criteria and who had just been fitted with hearing aids were then invited to attend an initial study session at the National Institute for Health Research (NIHR) Nottingham Biomedical Research Centre (BRC). The time duration between hearing aid fitting and attending the first study session was, on average, 5.25 days (SD= 4.81). Informed written consent was obtained prior to the start of this session. Outcome measures were obtained and then participants were shown m2Hear and given some instruction on how to use it. The participants were asked to use m2Hear on their own devices in their everyday lives as and when they needed, and were encouraged to use as much of the content that they thought was relevant to them. Following a period of independent use (M = 10.55 weeks, SD= 0.96), participants attended a second study session where outcome measures were obtained. As per Terwee et al. (2007), 50 participants were required to allow for sufficient between- and within-subject variability with regards to responses on the Clinical Global Impression Scale, described below. Attrition, based on our previous studies, was set at 18%. Therefore, a total of 59 participants were recruited. Hearing loss was measured using pure-tone air-conduction thresholds measured at octave frequencies (0.25–8 kHz), following the recommended procedure of the British Society of Audiology (2011).

All participants were each paid a nominal inconvenience allowance and travel expenses. The study was approved by the NHS Health Research Authority, East of England – Cambridgeshire and Hertfordshire Research Ethics Committee and Nottingham University Hospitals NHS Trust Research and Innovation department.

## Intervention

The m2Hear intervention (https://www.nottingham.ac.uk/helm/dev-test/m2hear/) was based on the original C2Hear multimedia educational programme and is described elsewhere (Ferguson et al, 2019a; Maidment et al. 2020b). In brief, m2Hear comprises:

- *Individualised earmould coupling*. Options for either open fits or custom earmoulds.
- *High-level categories (n = 5).* The mRLOs were divided into categories relating to the likely need along the patient journey post-fitting (i.e. using your hearing aids; getting used to your hearing aids; looking after your hearing aids; communication with others; using phones and other devices).
- *Series of 42 interactive mRLOs*. Each has a specific user-centred question (e.g. *How do I know which hearing aid is for my left/right ear?*), which were between 20s and 1min 56s duration (average = 60s).
- *Activities* (n = 6). These were associated with specific mRLOs (e.g. drag and drop activities: (i) to label the different components of a hearing aid, and (ii) how to work with others to help take part in conversations). Additional feedback was provided, including whether the chosen selection was correct or not, and why. These activities can be used as many times as necessary.
- *Interactive quiz* (n = 34 questions). These incorporated feedback to inform whether the questions were answered correctly or incorrectly, with additional supporting information relevant to the specific situation.

In summary, individualised learning opportunities were provided and the user had complete freedom and choice as to what they viewed according to their own specific listening, communication, and hearing aid needs. As the platform enabled users to actively engage in a range of learning activities, interactivity was also increased.

## Hearing aids

Hearing aids (Oticon Spirit Zest or Phonak Nathos S + Micro) were fitted using the NAL-NL2 algorithm and verified by real-ear measurements according to local protocols and national guidelines. Hearing aids were fitted with either open-fit slim tubes (78%) or custom earmoulds (22%), and 73% were bilateral fits.

## Outcome measures

### Self-report questionnaires

The outcomes were selected based on the World Health Organisation’s International Classification of Functioning, Disability and Health (ICF) (WHO 2001) that provides a theoretical framework upon which to measure the success of amplification using hearing aids (Granberg et al. 2014; Ferguson et al. 2017). All questionnaires were completed by interview at the baseline post-fitting (as unaided) and follow-up sessions (as aided).

#### Activity limitations/participation restrictions

The *Glasgow Hearing Aid Benefit Profile (GHABP)* (Gatehouse 1999) assesses unaided activity limitations (hearing disability) and participation restrictions (hearing handicap) (Part I), which was assessed during the first study session. Hearing aid use, benefit, residual disability, and satisfaction (Part II) was assessed at the follow-up session. Each of the domains is measured across four predefined situations and scored using a five-point scale, which are averaged and then converted to a percentage.

The *Hearing Handicap Inventory for the Elderly (HHIE)* (Ventry and Weinstein 1982) is a 25-item questionnaire that assesses the effects of hearing loss on emotional (*n* = 13), and social/situational (*n* = 12) domains of older adults. It is scored on a three-point scale (4 = yes; 2 = sometimes; 0 = no). The HHIE is not a unidimensional scale in its current form (Heffernan, Weinstein, and Ferguson 2020).

The *Social Participation Restrictions Questionnaire (SPaRQ)* (Heffernan, Coulson, and Ferguson 2018; Heffernan et al. 2019) is a 19 item measure that has two subscales, social behaviours (*n* = 9) and social perceptions (*n* = 10). It is scored on an 11-point scale (0 = completely disagree; 10 = completely agree). The SPaRQ is not a unidimensional scale, although both subscales are unidimensional.

#### Personal factors

The *Measure of Audiologic Rehabilitation Self-efficacy for Hearing Aids (MARS-HA)* (West and Smith 2007) is a 24-item questionnaire that assesses hearing aid self-efficacy across four subscales (basic, *n* = 7; advanced hearing aid handling, *n* = 5; adjustment to hearing aids, *n* = 3; aided listening, *n* = 9). Each item is scored on an 11-point percentage scale (0%=cannot do this at all; 100%=certain I can do this), with a mean score to give a global self-efficacy score.

The *Hearing Aid and Communication Knowledge (HACK)* (Ferguson et al. 2015) is a 20-item open-ended questionnaire targeting free recall of practical (*n* = 12) and psychosocial factors (*n* = 8) relating to hearing aid use and communication. A pre-defined marking scheme was used to allocate one point for every correct response, with a maximum capped score. The scores were totalled for each subscale, and the total of the subscales was converted to a percentage score to give a overall knowledge score.

The *Clinical Global Impression Scale* is a one-item question that asks, “All things considered, how is your overall hearing difficulty now, compared to before you started using the intervention?”. This was used to identify the minimally important change (MIC) for m2Hear when used with hearing aids. Responses were rated on a seven-point scale (*much improved* to *much worse*).

*IT literacy* was assessed using a validated three point scale for PC use (*never used a compute*r, *beginner*, *competent*) (Henshaw et al. 2012).

#### Functional measures

*Hearing aid use* (average hours/day) using data logging information from the hearing aids was obtained for the period between the fitting and evaluation sessions.

The *Montreal Cognitive Assessment* (MOCA) (Nasreddine et al. 2005) is a validated screening assessment for mild cognitive impairment assessing several domains including short-term memory, visuospatial abilities, executive function, attention, language abilities, and orientation. Total scores range from zero to 30 points, with higher scores indicating better cognitive function. A score ≥26 is considered “normal”.

#### Participant feedback

A *feedback questionnaire* was adapted from the one used in the original C2Hear RCT (Ferguson et al. 2016). There are 18 statements and participants were asked to rate their agreement on a five-point Likert scale (1 = strongly disagree to 5 = strongly agree). Each question tapped into one of three components relating to the feedback, which were content, design and consequences of using m2Hear. The questionnaire also included the following four optional open-ended questions: (i) *What was the worst aspect(s) of your experience with m2Hear?* (ii) *What was the most useful aspect of m2Hear?* (iii) *What did you learn from m2Hear that you used in your everyday life?* and (iv) *What would you change to make m2Hear more interesting, enjoyable or engaging?*

The *System Usability Scale* (SUS) (Sauro 2011) is a 10-item questionnaire that provides a measure of subjective assessment of usability of products and services, where each item is measured on a 5-point Likert scale, ranging from *strongly disagree* to *strongly agree*. Scores for each item range from 0 to 4. A composite score, ranging from 0 to 100, is obtained by multiplying the sum of all item scores by 2.5. A score greater ≥68 is considered “above average”, and anything less than 68 is “below average”.

The *Mobile Application Rating Scale: user version* (uMARS) (Stoyanov et al. 2016) is a questionnaire that assesses the quality of m-health applications from the user’s perspective. For overall quality, 16-items are measured on a five-point scale across four subscales (engagement, functionality, aesthetics, information). An additional four items assess subjective quality, also measured on a five-point scale.

*Semi-structured interviews* (*n* = 16) were held following the evaluation session with DWM, duration approximately one-hour. The transcripts were analysed using an established deductive thematic analysis procedure underpinned by the COM-B model (Maidment et al. 2020a).

### Analysis

Descriptive statistics (mean, standard deviation) were used to describe the characteristics of the sample, as well as the SUS and uMARS and outcome measures obtained at baseline (V1) and follow-up (V2). Planned statistical analysis to assess any change in the outcome measures between V1 and V2 was the paired *t*-test. Bonferonni correction was applied to outcome measures that had multiple subscales. Effect size (Cohen’s *d*) was categorised as small (0.2), moderate (0.5) and large (0.8). Significance was set to *p* ≤ .05.

## Results

### Participants

Of the 59 first-time hearing aid users who consented and attended the first baseline post-fitting session (V1), 52 (88.2%) participants attended the second follow-up session (V2, mean = 11 weeks post-fitting). Attrition was lower than expected, with seven participants dropping out (11.8% attrition), compared to our estimate of 18%. Reasons offered were not intervention-specific (illness *n* = 3, no reason offered *n* = 3, family bereavement *n* = 1). Participant demographics for both sessions are shown in Table 1. There was no significant difference in the demographics between the participants at V1 and V2, indicating that despite dropouts the participants seen at V2 were representative of those seen at V1.

**Table 1.** Demographics of the participants at baseline (V1) and follow-up (V2) session, showing the mean or number (n), and either SD or % in brackets.

	V1 baseline immediately post-fitting (n = 59)	V2 follow-up 10–12 weeks post-fitting (n = 52)
Age (years)	65.3 (13.1)	65.2 (13.1)
Age range (years)	29–90	29–90
Sex, female	32 (54.2%)	28 (53.8%)
Better ear average_0.25–4kHz _(dB HL)	24.7 (9.4)	23.7 (8.5))
GHABP Disability (%)	41.6 (16.4)	42.6 (15.2)
No. Hearing aids, bilateral	43 (72.9%)	37 (71.2%)
Earmould type, open	46 (78%)	43 (82.7%)
Montreal Cognitive Assessment	24 (1.4)	24 (1.4)
Lives with others (n)		
Yes	48 (81.4%)	42 (80.8%)
No	11 (18.6%)	10 (19.2%)
IT literacy: Computer skill (n)		
Competent	51 (86.4%)	45 (86.5%)
Beginner	8 (13.6%)	7 (13.5%)
Devices used (n)		
Smartphone	47 (79.7%)	42 (80.8%)
Tablet	46 (76.3%)	38 (73.1%)
Laptop	37 (62.7%)	32 (61.5%)
PC	31 (52.5%)	29 (55.8%)

### Digital literacy

Digital literacy was high with the majority (86%) considering themselves IT literate. This is also reflected in the high use of devices, with 80% using smartphones, consistent with other reports (Deloitte 2019), and 86% owning at least one other device. Mobile devices were used regularly, at least 2–3 times per day (smartphone = 88%; tablet= 63%), and their use was higher than for laptop or desktop PCs. Eight participants did not have a laptop or desktop PC, and only four did not have a smartphone or tablet. This almost certainly reflects the fact that the participant needed to meet the inclusion criteria for smartphone use.

### m2Hear: delivery, accessibility, usability, adherence

All device delivery options were used to deliver and access m2Hear on the participants’ own devices. Mobile devices (smartphone, tablets) were used by 50%, with the most common being tablets (42.3%) (Table 2). Similarly, the primary device used to access m2Hear was tablets (38.5%), although 6% used smartphones, and 55% used either a laptop or PC. This suggests that all these platforms are acceptable to first-time hearing aid users, and although multiple devices were often used, there remained a spread of device use.

**Table 2.** Use of smartphones, tablets, laptops and PCs to deliver and access m2Hear.

Total use (n = 52)	Primary device used	Additional use of technology		
		Tablet	Laptop	PC
Smartphone (12; 23.1%)	3 (5.8%)	8	2	5
Tablet (22; 42.3%)	20 (38.5%)	–	3	4
Laptop (20; 38.4%)	17 (32.7%)		–	3
PC (18; 34.6%)	12 (23.1%)			–

In terms of accessibility, all participants visited the m2Hear site at least once, with total of 178 recorded sessions. Two-thirds (65%) of devices that accessed m2Hear visited on 2+ occasions, and half (51%) on 3+ occasions. These return visits were higher than reported for C2Hear, although session duration was shorter (mean = 11mins 42 s), and individuals accessed m2Hear on more occasions. Unfortunately, due to a technical problem, it was not possible to identify the number of times each mRLO was accessed. However, participants reported that they revisited the m2Hear mRLOs multiple times because they were shorter. In terms of patterns of use, participants reported revisiting the mRLOs across sessions for reminders of information. Activities were accessed multiple times within a session, but less so across sessions because once completed there was little need to revisit for reminders. The activities that were used the most were those that addressed practical knowledge and insertion, which were accessed at least once for one-third of sessions (39.3% and 33.1%). The communication activities were accessed at least once in 22.4% of cases.

Usability, as measured by the SUS was high at 88.5% (SD= 6.6), where a score ≥68 is considered as average. Usability was also high when measured by the uMARS, shown in Table 3. The uMARS subscale for Information showed the highest ratings (M = 4.65), indicating high quality and informational content from credible resources. Functionality was also high (M = 4.46) with m2Hear having high performance of features and being easy-to-use, likely reflecting the high levels of user-involvement in the development. The Aesthetics were also rated well (M = 4.22), with the layout and quality of graphics rated highly (>4.3), although visual appeal was rated less highly (M = 3.9). Engagement was rated the lowest of all the subscales (M = 3.61). Despite m2Hear having a focus on being more interactive, the uMARS scores relate to customisation and prompts, and for m2Hear there was little the user could change about the actual design of the platform interface. However, the items relating to Interest (M = 4.2) and Target audience (M = 4.5) were rated highly, again likely reflecting the user-centred nature of the m2Hear development. The items Customisation (M = 3.1) and User input (M = 2.83) were rated the lowest of all the items because as noted above, m2Hear was limited in terms of how the user could change about the actual design of the platform interface.

**Table 3.** Mean, standard deviation, minimum and maximum values for the scales and subscales of the Mobile Application Rating Scale:users version (uMARS).

	Mean	SD	Min	Max
**Information**	**4.65**	**.05**	**2**	**5**
Quality of information	4.47	.67	2	5
Quantity of information (concise, comprehensive)	4.65	.56	3	5
Visual information (concepts, images)	4.57	.50	4	5
Credibility of resources	4.88	.38	3	5
**Functionality**	**4.46**	**.06**	**3**	**5**
Performance of features and components	4.42	.78	3	5
Ease of use	4.76	.52	3	5
Navigation between screens	4.23	.65	3	5
Gestural design (e.g. taps/swipes)	4.46	.54	3	5
**Aesthetic**s	**4.22**	**.70**	**3**	**5**
Layout and size (buttons content) is appropriate	4.41	.70	3	5
Quality of graphics is high	4.35	.74	3	5
Visual appeal	3.90	.41	3	5
**Engagement**	**3.61**	**.08**	**1**	**5**
Fun/entertaining to use	3.48	.70	1	5
Interesting to use	4.19	.79	2	5
Customise settings/preferences	3.10	1.16	1	5
Allows user input, provides feedback/prompts	2.83	1.25	1	5
Appropriate for target audience	4.50	.58	3	5

In terms of adherence, the participants were instructed to use the mRLOs however they wanted. The majority reported they watched all mRLOs (55.7%), and a third (36.5%) watched when they required specific information. A further 32.6% reported they watched the mRLOs regularly (e.g. 2–3 times a week), and a small proportion (7.7%) watched only those that seemed most relevant within the first 1–2 weeks. Participants’ use of mRLOs was varied, as expected, depending on their personal needs/preferences.

### Participant feedback

Participant views on m2Hear were sought by asking 18 closed statements. Table 4 shows the number and percentage of participants that agreed, disagreed or neither agreed or disagreed with the statements, ranked by positivity. All statements indicated that the majority of participants were positive about m2Hear. Seven statements showed >90% agreement in positive statements about m2Hear, which focussed mainly on content and design (e.g. *the videos were pitched at the right level* (92%), *I liked that the videos were short in duration* (98%)). This is consistent with the usability results. Interestingly, 5 of the 12 statements that were rated as >80% agreement, indicated that m2Hear had resulted in positive consequences for the participants (e.g. *If I had a problem with my hearing or hearing aid I would refer back to m2Hear* (92%)).

**Table 4.** Participant’s feedback on m2Hear.

Statement	Strongly Disagree or Disagree n (%)	Neither Disagree or Disagree n (%)	Strongly Agree or Agree n (%)
The videos aided my understanding of the topics	0 (0%)	1 (2%)	49 (98%)
I liked that the videos were short in duration	1 (2%)	0 (0%)	49 (98%)
I found m2Hear difficult to use	48 (96%)	0 (0%)	2 (4%)
It was important to me to be able to select which videos to view	0 (0%)	3 (6%)	47 (94%)
If a problem with my hearing or hearing aid arose, I would refer back to m2Hear	2 (4%)	2 (4%)	46 (92%)
The videos were pitched at the right level	2 (4%)	2 (4%)	46 (92%)
I liked that the order of the topics fitted around when I might need to use them (e.g. change batteries first, communicate with others later)	5 (10%)	0 (0%)	45 (90%)
I used m2Hear because it might make me hear and communicate better	(4%)	4 (8%)	44 (88%)
The videos held my interest	7 (14%)	0 (0%)	43 (86%)
If I had a question about hearing aids I was able to find the answers easily	3 (6%)	5 (10%)	42 (84%)
m2Hear has given me more confidence to use my hearing aids and communicate with others	1 (2%)	7 (14%)	4 (84%)
m2Hear did not provide me with any additional information to the advice given to me by my audiologist	41 (82%)	4 (8%)	5 (10%)
I felt motivated to use m2Hear	3 (6%)	8 (16%)	39 (78%)
The quizzes and activities gave me clear messages in understanding what is right and what is wrong	2 (4%)	11 (22%)	36 (74%)
I would prefer written information than use m2Hear	35 (70%)	11 (22%)	4 (8%)
The quizzes and activities were valuable in showing me what I had learned	1 (2%)	15 (31%)	33 (67%)
Now that I have used m2Hear I am more likely to contact audiology if I have a problem	8 (16%)	11 (22%)	31 (62%)
It was important to me that m2Hear could be tailored to my needs and preferences	5 (10%)	15 (31%)	29 (59%)

A summary of the responses to the open-ended questions is shown in Table 5. The most useful aspect reported by participants was that m2Hear increased knowledge of hearing aid handling, maintenance and communication (*n* = 19), followed by being accessible and convenient to use (*n* = 8), and being clear, concise and comprehensive (*n* = 8). Some of this was reflected in terms of what individuals had learned, which they could use in their everyday life. There were equal responses to practical hearing aid handling and maintenance skills (*n* = 14), and how to communicate successfully with others (*n* = 14), with learnings also for persevering and acclimatising to hearing aids (*n* = 5). The highest number of reports for the worst aspects of using m2Hear were for “nothing” (*n* = 13) and initial navigation and/or orientation issues when first using m2Hear (*n* = 13), such as being unsure how to go back to previous content or where to click to access an mRLO. Some participants also reported that the worst aspect was that the hearing aid featured in some of the mRLOs was dissimilar to the model that had been fitted with (*n* = 8). In terms of what the participants reported they would like to change to make m2Hear more interesting, enjoyable or engaging, the highest number of comments was for “nothing” (*n* = 18). Nevertheless, some participants requested that they would have liked less content focussed primarily at older adults (*n* = 5), and would like additional content on the technical aspects of hearing loss and how hearing aids work (*n* = 4).

**Table 5.** Open-ended participant feedback about m2Hear and number of reports (n).

What was the most useful aspect of m2Hear?	*n*	What did you learn that you used in your everyday life?	*n*	What was the worst aspect of m2Hear?	*n*	What would you change?	*n*
Increased knowledge of HA handling/maintenance and communication	19	Practical hearing aid handling/maintenance skills	14	Nothing	13	Nothing	18
Accessible and convenient to use	8	How to communicate with others	14	Initial navigation/orientation	13	Less content aimed at primarily older adults	5
Videos clear, concise and comprehensive	8	Value of perseverance and acclimatisation of hearing aids	5	Difference between model of hearing aid and that featured in videos	8	More technical information about hearing aids and hearing loss	4
Provided useful reminders	5	Troubleshooting	3	Quiz questions unclear/ambiguous	3	Develop as a standalone app rather than web browser	2
Provided reassurance and confidence	4	How to use phone and other devices (e.g. loop)	3	Screen size too small on smartphones	2	Improve visual appeal of interface/content	2
Facilitated independence/self-management	2			Images only include older adults	0	More relevant /comprehensive quiz questions	2

### Outcome measures

**Figure 1.**
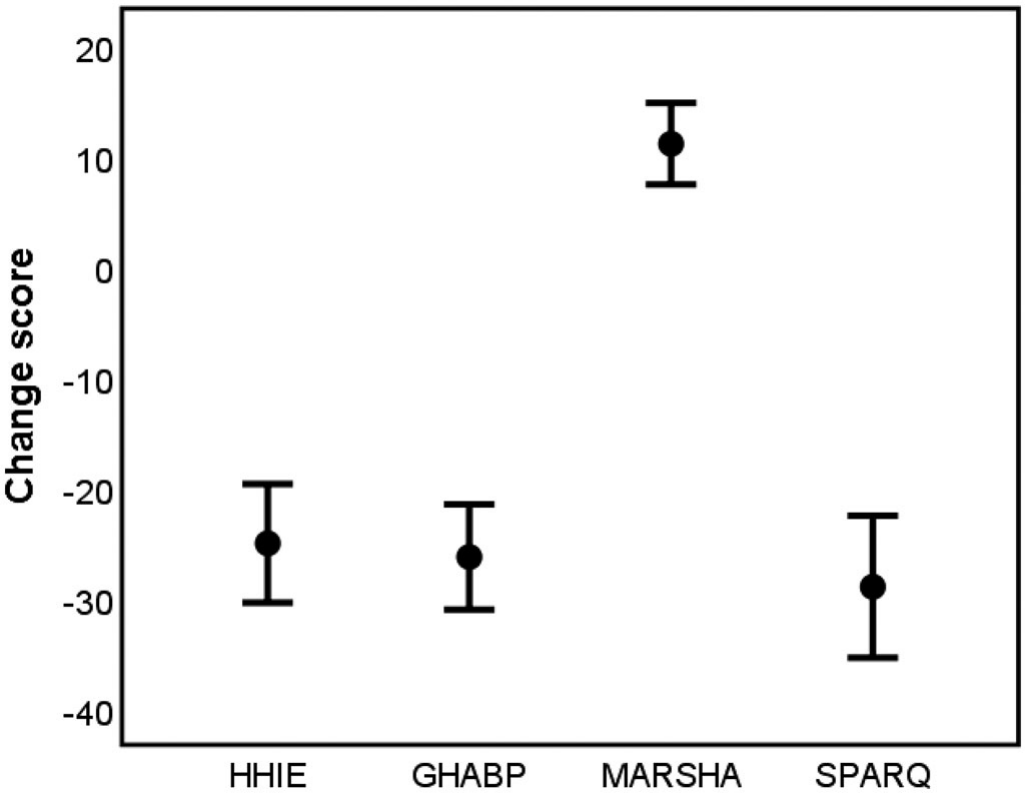
The mean and 95% CI for the change score between V2 (follow-up) and V1 (baseline) for the outcome measures. HHIE: Hearing Handicap Inventory for the Elderly; GHABP: Glasgow Hearing Aid Benefit Profile; MARS-HA: Measure of Audiologic Rehabilitation Self-efficacy for Hearing Aids; SPaRQ: Social Participation Restrictions Questionnaire.

The mean and 95% confidence intervals for V1 and V2 outcomes and subscales are shown in Table 6, and the V2–V1 difference score for key outcomes are shown in Figure 1. For all the outcome measures and their subscales (adjusted for multiple comparisons using Bonferroni correction), there was a significant improvement between the post-hearing aid fitting and the follow-up sessions as a result of the hearing aid and m2Hear. All showed large effect sizes.

**Table 6.** Mean and SD (in brackets) for each of the outcome measures overall and subscale scores at the V1 baseline post-fitting and V2 follow-up sessions.

Outcome	V1 Baseline post-fitting	V2 10 weeks follow-up	*t*	*p* Value	*d*
HHIE Overall	33.6 (19.2)	10.2 (1.5)	8.9	<.001	1.7
Emotion	16.2 (11.5)	4.7 (6.7)	7.9	<.001	1.2
Situation	17.4 (8.8)	5.5 (4.9)	9.1	<.001	1.7
GHABP					
Hearing disability	42.4 (15.3)	17.4 (13.2)	10.5	<.001	1.7
SPARQ					
Behaviour	38.5 (19.8)	10.5 (15.8)	9.6	<.001	1.6
Perception	38.4 (11.9)	11.9 (16.5)	8.0	<.001	1.8
MARSHA Overall	79.9 (12.1)	90.7 (6.7)	6.2	<.001	1.1
Basic handling	88.6 (14.0)	98.6 (2.4)	5.0	<.001	1.0
Advanced handling	52.3 (23.8)	76.0 (18.0)	6.6	<.001	1.1
Adjustment	85.1 (18.0)	93.0 (6.8)	2.6	.013	0.6
Aided Listening	86.8 (12.4)	92.1 (6.8)	3.0	.005	0.5
HACK Overall	41.1 (7.1)	50.8 (7.7)	8.0	<.001	1.3
Practical	42.3 (8.0)	53.0 (8.4)	7.0	<.001	1.3
Psychosocial	39.6 (10.3)	48.1 (10.6)	5.8	<.001	0.8
Datalogging (hours/day)	–	9.5 (3.9)	–	–	–

Effect size is shown by Cohen’s *d*. HHIE: Hearing Handicap Inventory for the Elderly; GHABP: Glasgow Hearing Aid Benefit Profile; SPARQ: Social Participation Restrictions Questionnaire; MARS-HA: Measure of Audiologic Rehabilitation Self-efficacy for Hearing Aids; HACK: Hearing Aid and Communication Knowledge questionnaire.

One of the objectives of this study was to identify which outcome measures would be suitable for use as a primary outcome measure in a future clinical effectiveness trial. As part of the decision making, we intended to assess meaningful change from the patient’s perspective, classifying patients according to their responses on the CGI scale. In accordance with Terwee et al. (2007), this is achieved by first calculating the mean change (i.e. difference) in scores for each outcome measure between V1 and V2. To identify the minimal important change (MIC) values, the difference between the mean change scores for “no change” and “improved” and for “no change” and “worse” CGI categories was then calculated. However, MIC values could not be obtained in the current study as the majority of participants (90.4%) responded at V2 that their hearing had “improved” compared to before using the intervention (i.e. hearing aids + m2Hear). The remaining participants responded, “no change”, with no participants reporting that their hearing was “worse”. Thus, we were unable to identify the minimal clinically important difference for hearing aids used in conjunction with m2hear. 

## Discussion

There is an increasing use of e- and m-health technologies in healthcare generally, and the same is true in hearing healthcare. Since the systematic review of Swanepoel and Hall (2010), there have been numerous developments which have been used to screen, diagnose, manage and support children and adults living with hearing loss. Among the many different terminologies used, an umbrella term for this is connected hearing healthcare. Some advantages of connected hearing healthcare help overcome barriers such as time, mobility, and geography by increasing accessibility and convenience to hearing healthcare. Other advantages include enabling personalised and tailored information to meet individual’s needs, as well as providing greater interactivity and control of resources thereby increasing engagement of individuals in their own hearing healthcare.

In this feasibility study, our focus was to assess these benefits, alongside aspects of delivery and usability, of the m-health educational m2Hear programme. In addition, we aimed to identify appropriate outcome measures for use in the evaluation of m2Hear in any future randomised controlled trials, in accordance with the MRC Guidance on developing and evaluating complex interventions (Craig et al. 2008). This is one of three papers, of which the other two describe the development of m2Hear (Maidment et al. 2020b), and the results from a qualitative analysis to examine which aspects of behaviour present as barriers and facilitators for the use of m2Hear in hearing aid users (Maidment et al. 2020a).

In this study, m2Hear was delivered mainly by mobile technologies (i.e. smartphones or tablets), with tablets being the primary mobile device used. This was because it was easier to read and engage with a tablet, although some participants did report that they also used their smartphones to access m2Hear “on-the-go”. Laptop use was, however, a close second to tablets. The range of devices used as the primary device, and multiple use of secondary devices, indicates that in this sample recruited from a typical UK NHS audiology clinic, all options are relevant. A subtheme of the qualitative study was digital literacy skills associated with an individual’s capability to use m2Hear (Maidment et al. 2020a). This was not an unexpected outcome as we have seen this in all our other studies on m-health interventions (Maidment, Ali, and Ferguson 2019; Gomez et al., accepted). Older adults often perceive their technological skills as being far inferior to others, seen in other hearing-related studies (Ng et al. 2017; Keidser, Matthews, and Convery 2019), and more generally (Kuerbis et al. 2017; Vaportzis, Giatsi Clausen, and Gow 2017). Whilst there are clearly differences in digital literacy between older and younger adults, often known as the “digital divide” (which can also include other reasons such as social and geographical), it is clear from recent research that a significant number of older adults are “tech-savvy”, and have the necessary skills to operate m-health interventions. Therefore, to exclude older adults from accessing digital and/or remote hearing technologies just because of their age is a disservice to this population, particularly as many can benefit from such technologies. The use of evidence-based behaviour change techniques by audiologists could support patients to adopt and successfully use these technologies to self-manage their hearing loss (Gomez et al., accepted).

Unlike the previous C2Hear studies where we asked the participants to watch all the RLOs at least once, in this study we were interested to see how the participants used m2Hear in their daily lives. All participants visited the m2Hear website at least once. Around two-thirds watched m2Hear at 2+ times, and half participants watched m2Hear 3+ times. The return use to m2Hear was higher than that seen for C2Hear, however the total duration of views for m2Hear was shorter than for C2Hear. This was how we had anticipated that m2Hear would be used (i.e. more regularly but for less time), as we left it to the individual to use m2Hear as and when they wanted to, in terms of seeking the information they needed, as and when required. This aligned well with the qualitative study where participants reported that m2Hear was convenient to use and provided useful reminders to help understand how hearing aids can be used and communication improved. The ease of use and conciseness of m2Hear, and the ability of the participants to choose which information they needed by homing in on the questions that were linked to each mRLO, provided a more personalised approach to meet their individual informational needs. Although we were unable to identify which mRLOs were watched and when, due to a technical problem, we saw that the practical activities (such as how to clean the tubing, and insert the hearing aid) were the most commonly completed (between 33 and 39%), whereas the communication activities (e.g. where to sit in a restaurant) were completed in about a fifth of cases. This was similar to how C2Hear was used in the original RCT, where there was a greater focus on practical rather than psychosocial aspects. However, we saw that in the open-ended questions, which asked about what people had learned in their everyday life, both practical hearing aid handling skills and communication were rated most highly, and equally so. This suggests that when people did watch the communication mRLOs, what they learned was both useful and used.

The usability of m2Hear was generally rated as being very good. In particular, the items relating to the Information subscale from the uMARS were all rated very highly (M = 4.65). As providing high-quality and relevant information was the main purpose of C2Hear when it was first developed, these usability scores indicate that the information embedded in m2Hear is doing what we originally set out to do. This aligns well with the analysis of the determinants of behaviour based on the COM-B and TDF, where capability in terms of knowledge and skills were the domains that were most evident in the materials (Maidment et al. 2020b). We also obtained uMARS scores relating to C2Hear in the study by Maidment et al. (2020b) and the Information subscale score (M = 4.5) was similar to that for m2Hear. Given the RLO material in both C2Hear and m2Hear are basically the same, this was not a surprise, albeit reassuring. For all the other uMARS scales, however, C2Hear was rated less well. So, where we see the Functionality subscale for m2Hear was high (M = 4.46), the overall Functionality rating for C2Hear was lower (M = 3.75). This difference was even more marked for the Aesthetics and Engagement subscales with m2Hear (4.22 and 3.61 respectively) rated higher than C2Hear (3.13 and 3.00 respectively). These scores reflect the interviews reported in Maidment et al. (2020a) for users of both m2Hear and C2Hear. m2Hear was reported to be more concise and more easily digestible than C2Hear, and the shorter mRLOs provided more specific personalised information, more readily. Overall, m2Hear was seen to be more usable than C2Hear. It is likely that the substantial involvement of hearing aid users in the participatory approach to their co-design and development helped to enhance usability more generally by aligning to hearing aid users’ needs.

All the self-report outcome measures showed a significant improvement between the time just after hearing aid fitting (V1, unaided) and the 10-week follow-up (V2, aided), with generally large effect sizes. As the intervention was the hearing aids plus m2Hear, with no control group who used hearing aids only, we were not able to evaluate m2Hear separately. However, due to the nature of this feasibility study the aim was not to evaluate the effectiveness of m2Hear *per se*, but to establish which outcome measures might be used in any future RCT, in particular, as a primary outcome measure. The largest effect sizes were seen for the HHIE, GHABP and SPaRQ (≥1.6), which all tap into activity limitations or participation restrictions. As improving participation, rather than improving listening to a talker (i.e. activity), is the main goal of auditory rehabilitation (Ferguson et al. 2019b), we would recommend the HHIE or SPaRQ as a primary outcome measure. Furthermore, given that the SPaRQ has been validated using modern psychometric methods (Heffernan, Coulson, and Ferguson 2018; Heffernan et al. 2019), and the HHIE has been shown not to be unidimensional, with a number items not fitting the 3-point response scale and having poor fit and/or differential item functioning (Heffernan, Weinstein, and Ferguson 2020), we would recommend using the SPARQ as a primary outcome measure in future RCTs.

Outcomes for self-efficacy for hearing aids, and knowledge about hearing aids and communication, improved significantly with large effect sizes, which we have also demonstrated previously for C2hear (Gomez and Ferguson 2020). These results were also reflected in the qualitative study where themes relating to improved confidence and empowerment were revealed (Maidment et al. 2020a). As both self-efficacy and knowledge have been identified as modifiable factors in self-management (Convery et al. 2019), and knowledge is a core dimension in empowerment, then there are opportunities for audiologists to promote both self-management and empowerment in patients using tools such as m2Hear. Methods to facilitate this based on behaviour change techniques (BCTs) related to mechanisms of action via the COM-B and TDF (Cane, O’Connor, and Michie 2012; Michie et al. 2014) have already been considered for support and improve the use of smartphone-connected hearing aids in daily life (Gomez et al., accepted). BCTs such as social support (practical), information about social, environmental and emotional consequences, and instruction on how to perform a behaviour could all be addressed by m2Hear or m2Hear-like interventions.

**Figure 2.**
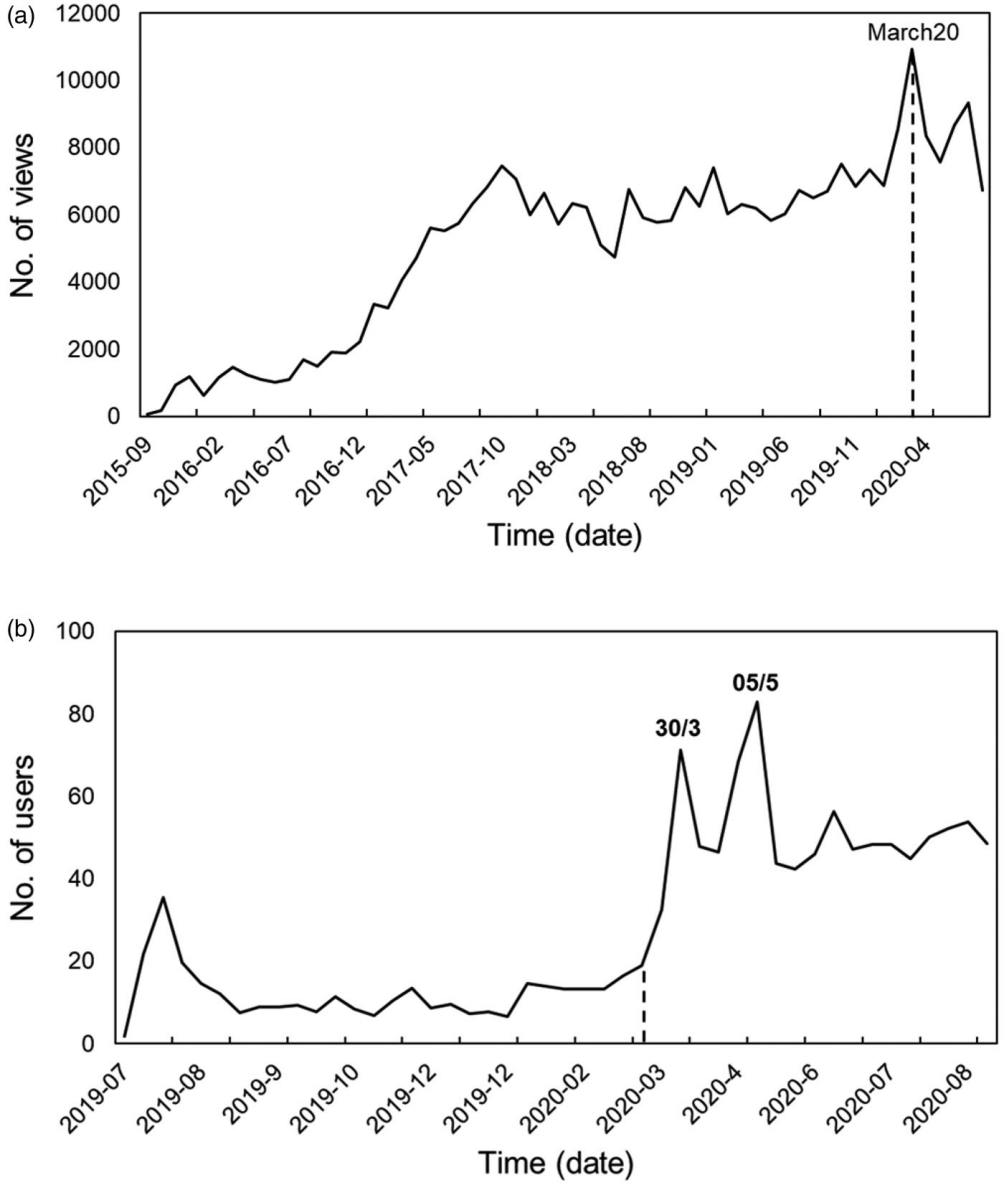
The number of (a) views for C2HearOnline YouTube, and (b) users of c2Hearonline.com, across time (year-month) finishing at the end of August 2020. The numbers on the peaks are the dates (e.g. 30/3 = 30th March 2020). The dotted line is the start of the COVID-19 pandemic in the UK at the point where there were 100 recorded cases.

In terms of the future of connected hearing healthcare, there has been a dramatic increase globally in the need to provide remote or hybrid (i.e. a combination of remote and personal) services to minimise face-to-face clinic appointments as a result of the COVID-19 pandemic. We have seen this for C2hear views also. Figure 2 shows that for both C2Hear platforms (You Tube www.youtube.com/C2HearOnline and the standalone website c2hearonline.com) there was a sharp rise in views around the time that COVID-19 infections were at 100 and starting to rise in the UK. Interestingly, the drop in the YouTube version in March 2020 coincided with an increase in the standalone version. In part, this may be because both c2Hearonline.com and m2Hear were included in the UK’s COVID-19 guidelines from the professional audiology groups (AIHHP, BAA, BSA, BSHAA, 2020), first published in March 2020. It is unlikely that the global increase in the use of connected hearing healthcare during the COVID-19 pandemic will return to previous levels as both audiologists and patients become aware and recognise the benefits of connected hearing healthcare. This also coincides in the same year as the United States Food and Drug Administration (FDA) specifications for regulations for the over-the counter (OTC) hearing aids come into force. A recently published Delphi review of UK hearing healthcare professionals (HCPs) reached a consensus that online information is essential for OTC devices that are fitted without HCP support (92%), such as C2Hear/m2Hear (88%) (Olson, Maidment, and Ferguson 2020).

Given the rapid growth of connected hearing healthcare in a relatively short period of several months, far exceeding what would have been expected prior to the pandemic, it is likely that the need for personalised information to improve the knowledge of hearing aids and communication will only increase. It can easily be envisioned that delivery of m-health interventions, such as m2Hear or m2Hear-like tools, could be delivered through a variety of routes. These may include apps, either standalone or as part of a hearing device system, or by integration with other technologies such as a virtual voice assistant (e.g. Alexa, Google Home). It is clear that m-health technologies offer new ways of providing and delivering (connected) hearing healthcare, and in this ever-changing landscape, the role of m2Hear and other m-health technologies are only likely to continue to rise.

## Study limitations

There were some study limitations. We were unable to identify which mRLOs were used and when due to a technical problem. This would have provided useful information to audiologists to help guide patients as to which mRLOs are likely to be the most helpful, and when. A second limitation was lack of outcome measures data to parse out the effectiveness of the incremental benefit of m2Hear over and above that of hearing aids. One way to address this would have been to ask the participants to complete the outcome questionnaires at V1. However, most participants would have only received their hearing aids a few days previously, so it was likely they would not have fully adjusted to their hearing aids. The inclusion of a control group would also have addressed this, although the purpose of this study design (i.e. to assess the feasibility of the intervention) did not support this.

## Conclusions

The feasibility study of 59 first-time hearing aid users showed that m2Hear was used most consistently with tablets, and around half of the sample used m2Hear with mobile technologies; the remainder used laptops/PCs. Usability was high, likely reflecting the user-centred co-production approach to develop m2Hear. Feedback on the use of m2Hear was very positive, with the vast majority reporting that the intervention was well-designed, enabling them to easily refer to relevant sections when they needed advice or answers to specific questions. m2Hear provides additional information and advice to that offered by the audiologist, suggesting that m2Hear would be an easy-to-use intervention to supplement clinical audiology practice. Outcome measures showed a range of benefits of using hearing aids alongside m2Hear, indicated by large clinical effect sizes. These included knowledge of hearing aids and communication, hearing aid self-efficacy, hearing difficulties, social engagement, and hearing hearing-related quality of life. We would recommend using the SPaRQ as the primary outcome measure in future trials of m2Hear. In conclusion, a theoretically-driven, personalised and co-designed educational m-health intervention (m2Hear) is feasible and beneficial for use in the self-management of hearing loss and hearing aids. With the rapid growth of connected hearing healthcare as a result of COVID-19, the relevance and use of m2Hear or m2Hear-like interventions is only like to increase in future.
